# Contraceptive Use and Reproductive Health in Women With CKD: A Qualitative Study of Nephrologists in the United States

**DOI:** 10.1053/j.ajkd.2025.07.007

**Published:** 2025-09-16

**Authors:** Nedas Semaska, Rachael Nolan, Silvi Shah

**Affiliations:** College of Medicine (NS), Department of Environmental and Public Health Sciences (RN), and Division of Nephrology, Department of Internal Medicine (SS), University of Cincinnati, Cincinnati, Ohio

## Abstract

**Rationale & Objective:**

Women with chronic kidney disease (CKD) face elevated risks during pregnancy, yet contraceptive use and reproductive health counseling remain low. Nephrologists, who often maintain longitudinal relationships with patients, may be well-positioned to engage in these discussions. This study explored nephrologists’ perspectives on contraception and reproductive health management in women with CKD.

**Study Design:**

Qualitative study using semistructured interviews.

**Setting & Participants:**

Interviews were conducted with 25 adult general and transplant nephrologists from both academic and private practice settings across the United States.

**Analytical Approach:**

Virtual interviews were recorded, transcribed, and analyzed using thematic analysis until thematic saturation was achieved. A grounded theory approach guided coding and identification of key themes related to provider experiences and perspectives.

**Results:**

The following 4 themes and their respective subthemes were identified: (1) physician discomfort regarding discussion of contraception and reproductive health (reliance on patient initiation, hesitation with counseling, uncertainty about scope of practice); (2) insufficient training and inadequate guidelines regarding contraception and reproductive health (paucity of formal guidelines, limited exposure, reliance on self-education); (3) lack of interdisciplinary coordination regarding contraceptive use and reproductive health (the patient as an intermediary, fragmentation of care); (4) need for holistic and patient-centered care (comprehensive and sustained approach, shared decision-making).

**Limitations:**

Generalizability may be limited due to participants being predominantly early-career academic nephrologists.

**Conclusions:**

Key barriers to contraceptive use and management of reproductive health for women with CKD include provider discomfort due to limited exposure and training, lack of clear guidelines, and fragmented care. Despite these challenges, providers recognize the importance of holistic, patient-centered care. These findings highlight the need to improve contraceptive counseling to support appropriate contraceptive use and shared decision making for the reproductive health of patients with kidney disease.

## Graphical Abstract

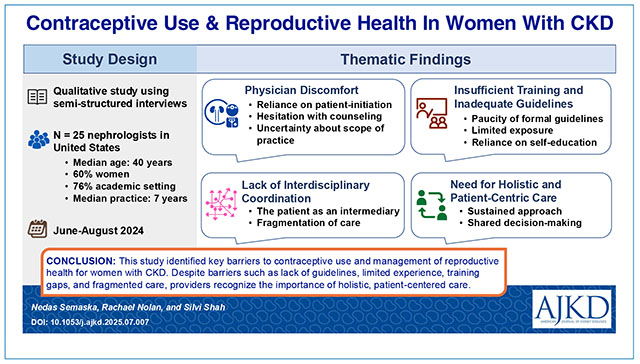

Chronic kidney disease (CKD) is a public health problem and impacts up to 6% of women in their childbearing years, although the true prevalence may be higher due to diagnostic challenges during pregnancy. Women with CKD face increased risks, including a 10-fold higher chance of developing pre-eclampsia and a 6-fold greater risk of preterm delivery compared with the general population.^1–4^ Furthermore, pregnancy after kidney disease is associated with the progression of CKD and is a risk factor for glomerular filtration rate (GFR) decline.^5,6^

CKD is associated with reduced fertility^7–9^; however, the landscape of reproductive health for women with kidney disease has evolved significantly. From 2002 to 2015, the number of deliveries among women undergoing dialysis and those who had received kidney transplants markedly increased.^10–12^ This trend is further underscored by the establishment of new pregnancy and kidney disease clinics, which are dedicated programs designed for women with kidney disease who are either pregnant or seeking to conceive.^13–16^ Despite this, the rate of contraceptive use in women with CKD remains low at less than 10%, and patients report a lack of adequate counseling and coordinated care, particularly regarding contraception and pregnancy management.^17,18^ The majority of US and Canadian nephrologists reported lacking confidence in managing women’s health issues, including menstrual disorders, preconception counseling, and pregnancy management.^19^

Although some nephrologists may view contraceptive counseling as outside the traditional scope of nephrology care, their longitudinal role makes them uniquely positioned to address reproductive health during routine clinical encounters. This is particularly critical in settings where care is fragmented or access to gynecologic or primary care services is limited. Integrating contraceptive counseling into nephrology aligns with emerging international and national best practices, including recommendations from the Kidney Disease Improving Global Outcomes (KDIGO)^20,21^ organization and the National Kidney Foundation (NKF),^22,23^ which advocate for a more holistic, patient-centered approach to CKD care.

Previous research has explored reproductive health in women with CKD by using quantitative physician surveys,^19,24^ qualitative patient interviews,^18,25,26^ and retrospective cohort studies.^27^ Recognizing the need for context-specific insights, qualitative interviews were chosen over quantitative methods because they provided nuanced insights into nephrologists’ subjective experiences and the factors that affect their decision making process. This qualitative study offers a comprehensive exploration of nephrologists’ beliefs and experiences regarding contraception and reproductive health in women with kidney disease.

## Methods

This study employed semistructured virtual interviews with a cohort of adult nephrologists across the United States. Ethics approval was obtained from the University of Cincinnati Office of Human Research Ethics (approval number: 2024-0345) and followed the Consolidated Criteria for Reporting Qualitative Health Research.^28^

### Interview Design and Content

Development of the interview guide was informed by a diverse body of literature addressing reproductive health in CKD, including physician survey studies,^19,29^ qualitative interviews,^18,25,30,31^ and a mixed-methods investigation.^26^ The physician surveys and interviews provided insight into provider knowledge gaps, counseling practices, and perceived barriers to discussing fertility and contraception with CKD patients. The patient qualitative studies illuminated the experiences of reproductive decision making, including uncertainty about fertility, concerns about pregnancy risks, and the emotional burden of navigating these issues in the context of chronic illness.

The interview guide underwent refinement through discussions within the research team and feedback from practicing nephrologists in the department. Specific issues considered during the development of the semistructured interview guide were item clarity, respondent burden, and alignment with predefined conceptual domains, which encompassed physician knowledge, attitudes, and behaviors regarding reproductive health in CKD patients. These considerations were established a priori, guided by best practices in interview guide design.^32^ To pilot the semistructured guide, 5 board-certified nephrology attending physicians—3 in general nephrology and 2 in transplant nephrology—were recruited for virtual interviews from 5 medical universities across the United States using convenience sampling methods. Pilot feedback was systematically evaluated, and the items identified as unclear, redundant, or misinterpreted were either revised or omitted. The minimal modifications made during the pilot phase did not necessitate discarding the pilot data, which was therefore included in the final analysis. The finalized interview template is provided in Item S1.

### Participant Selection

The study targeted nephrologists practicing in the United States with experience in managing women of childbearing age with CKD. Potential interviewees were identified through professional connections within the nephrology network, and those contacted were invited to refer additional qualified nephrologists, facilitating a snowball sampling method.^33^ Upon identification of a potential participant, an initial email invitation was sent, followed by a reminder email sent 7 days later. The emails contained information about the study’s purpose, interview details, and scheduling instructions. Informed consent was obtained via a web-based consent form. This study was approved by the Institutional Review Board at the University of Cincinnati.

### Data Collection

Interviews (n = 25) were conducted with nephrologists from across the United States. Thematic saturation was deemed to have been reached at that interview count, with no additional themes identified. The interviews used open-ended questions and lasted between 20 to 50 minutes. To enhance accessibility among a range of geographic regions, all interviews were conducted, recorded, and transcribed verbatim using Microsoft Teams software [version 24124.1412.2911.3341; Microsoft Corp].

### Data Analysis

After transcription, each transcript was promptly reviewed and manually corrected to ensure accuracy and remove any identifying information. Members of the research team (R.N. and N.S.) conducted thematic analyses on each transcript, collaborating with the principal investigator to identify key concepts and representative quotes. This process facilitated the development of an initial coding scheme to understand participants’ reasoning. Themes were derived inductively by applying a grounded theory approach.^34^

## Results

### Demographics

Interviews were successfully conducted with 25 nephrologists practicing in academic (76%), private (4%), and hybrid (20%) settings. The participants included both men (40%) and women (60%) who identified their clinical scope as either general nephrology (68%) or transplant nephrology (32%).

The majority of participants worked with adult patients (93%); 1 treated only pediatric patients (n = 1), and another treated both adult and pediatric populations (n = 1). The median age of the interviewed nephrologists was 40 years (IQR, 38-44), with an average of 9 years of practice since completion of fellowship. The demographics of the study participants are shown in Table 1.

### Themes

**Figure 1.**
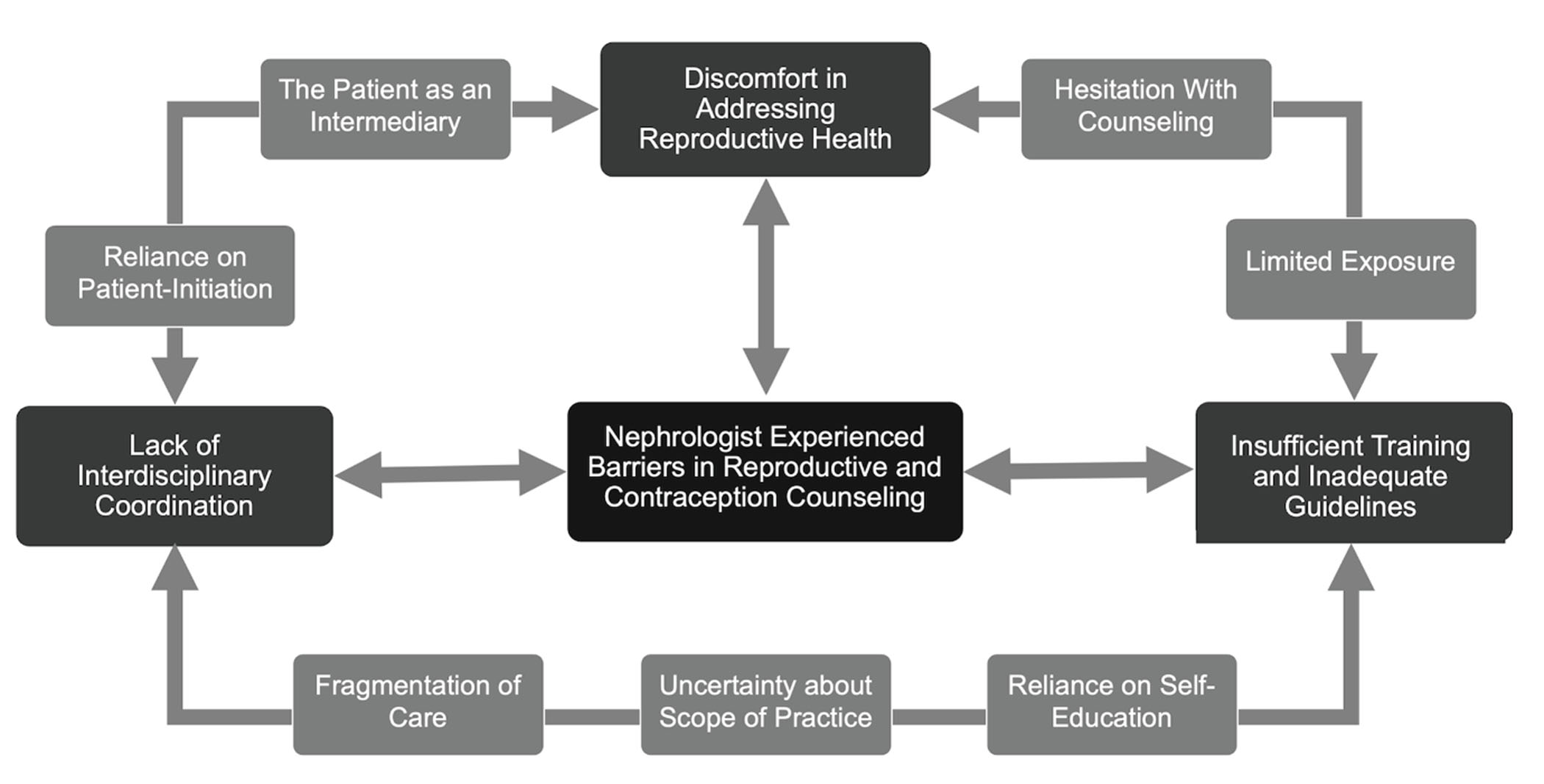
Thematic schema of relationship between barriers and nephrologists’ experiences providing contraception and reproductive health counseling.

Framework analysis of the interviews identified the following 4 major themes related to contraceptive use and reproductive health in women with kidney disease: (1) physician discomfort regarding discussion of contraception and reproductive health, (2) insufficient training and inadequate guidelines regarding contraception and reproductive health, (3) lack of interdisciplinary coordination regarding contraceptive use and reproductive health, and (4) need for holistic and patient-centric care. The themes and respective subthemes are described in the following sections, with illustrative quotations provided in Box 1. A thematic schema showing the patterns and relationships among themes and the attitudes and barriers of the participants is provided in Figure 1.

#### Theme 1: Physician Discomfort Regarding Discussion of Contraception and Reproductive Health

##### Reliance on Patient-Initiation.

Physicians reported waiting for patients to initiate discussions around reproductive health topics, including fertility, contraception, and pregnancy risks, in women with kidney disease. These topics were not routinely addressed in clinical visits, and nephrologists expressed low rates of reproductive counseling due to the topics not being explicitly raised by patients. By contrast, some nephrologists identified the prescription of teratogenic medications, such as angiotensin-converting enzyme (ACE) inhibitors or angiotensin II receptor blockers (ARBs), as opportunities to initiate conversations about reproductive health.

##### Hesitation With Counseling

A general hesitation with reproductive counseling was noted by the participants. This hesitation was attributed to limited encounters with patients of childbearing age, a lack of knowledge or confidence in managing reproductive health, and time constraints within clinical encounters. According to the interviewed physicians, the hesitation was not limited to clinicians but was also apparent among patients, contributing to the infrequency of these discussions.

##### Uncertainty About Scope of Practice.

Respondents expressed uncertainty about their role in contraceptive counseling, and some suggested that these conversations might be better managed by other physicians such as gynecologists or primary care physicians. Some reported nephrologists’ scope as being primarily centered on kidney-related issues, leaving broader reproductive health topics outside their expertise. However, some participants emphasized the role of nephrologists in ensuring that reproductive health counseling occurs even if they are not the ones providing the counseling.

#### Theme 2: Insufficient Training and Inadequate Guidelines Regarding Contraception and Reproductive Health

##### Paucity of Guidelines.

The participants reported a lack of established guidelines as a source of uncertainty in providing reproductive and contraceptive use counseling to women with CKD. The lack of standardized recommendations was noted to hinder decision making, particularly when tailoring counseling to individual patient circumstances. The respondents also highlighted gaps in the literature, including limited exploration of infertility correlations with varying degrees of kidney dysfunction and etiological differences.

##### Limited Exposure.

The relatively lower prevalence of CKD among women of reproductive age was stated as a contributing factor to nephrologists’ limited experience. Several respondents suggested that additional resources, such as educational sessions, could help address this experience gap. Additionally, some respondents believed that nephrologists’ knowledge regarding sexual and reproductive health in women with CKD might be outdated.

##### Reliance on Self-Education.

Providers highlighted the need for independent study due to minimal exposure to reproductive health topics during medical school, residency, and fellowship training. Many emphasized the importance of incorporating reproductive health education into nephrology training to sensitize providers to these issues. It was stated that if providers are not exposed to these topics earlier in their careers, they will remain unsensitized. Case method teaching was suggested as a possible route to engage providers with reproductive health.

#### Theme 3: Lack of Interdisciplinary Coordination Regarding Contraceptive Use and Reproductive Health

##### The Patient as an Intermediary.

Interviewed physicians described shared clinical notes as being the primary mode of communication between providers. However, uncertainty was expressed about whether these notes were consistently read by other specialists. As a result, nephrologists reported relying on patients to act as the intermediary between different specialists, often giving referral recommendations and expecting patients to proactively initiate contact with other providers.

##### Fragmentation of Care.

The coordination of reproductive health care for women with CKD was described as fragmented and challenging, particularly when providers operated in separate health care systems. Nephrologists noted that although referrals to obstetricians or gynecologists were commonly made, the patient might then be referred to yet another specialist. Often in situations where patients were stable, providers made assumptions that other specialists were managing their respective responsibilities. Additionally mentioned was the importance of having a “go-to” person, described as a trusted and knowledgeable provider with whom nephrologists could reliably communicate with when caring for complex patients.

#### Theme 4: Need for Holistic and Patient-Centric Care

##### Comprehensive and Sustained Approach.

Nephrologists noted that addressing both clinical outcomes and the emotional aspects of care contributed to a more holistic approach to reproductive health. The importance of ongoing and empathic conversations about the impact of CKD on reproductive health was highlighted. Some interviewed nephrologists stressed that these discussions should not be one-time interactions but rather recurrent conversations to reinforce patient understanding.

##### Shared Decision Making.

Nephrologists reported that building trust with patients played a crucial role in improving physician–patient communication. They emphasized that trust not only improved provider confidence in discussing sensitive topics but also empowered patients to engage with recommended resources and actively participate in decision making. Providers noted that even brief initiation of these conversations empowered patients to ask questions and seek additional information.

## Discussion

The nephrologists in our study expressed hesitation in initiating discussion about contraceptives and reproductive health. This stemmed from a variety of reasons, including limited exposure to women of childbearing age, it not being part of routine practice, or discomfort with the topic. This parallels findings in prior research, which revealed that women with CKD often avoid initiating such discussions due to fear of judgment or concerns about prioritizing pregnancy over their health.^30^ Although not directly explored in our study, participant discomfort may also reflect underlying personal or cultural beliefs, suggesting a need for future research into how provider values, US sociopolitical climate, and regional variation shape reproductive counseling.

Furthermore, another study highlighted patients’ frustration with inadequate reproductive counseling, with many women wishing these conversations had been initiated earlier.^18^ This hesitation on both sides of the patient–physician relationship highlights a communication gap that may contribute to the reported lower rates of contraceptive counseling in this population when compared with the general population.^26,35^ These counseling deficiencies extend to posttransplantation care, a critical period when fertility often returns.^27,36^ One study reported that 39% of women with kidney transplants were unaware of posttransplant pregnancy considerations,^37^ a notable proportion given that kidneys are the most commonly transplanted organ in women of reproductive age.^38^

Nephrologists are often perceived as primary care providers, particularly by dialysis patients.^39^ In conditions such as recurrent lupus nephritis and nephrotic syndrome, where frequent follow-up is required, women may seek pregnancy counseling from their nephrologist due to overlapping concerns ranging from immunosuppressive therapy to thrombosis risk and contraceptive safety.^40–42^ These scenarios illustrate how reproductive decision making intersects with kidney pathophysiology and pharmacologic management, reinforcing the need for nephrologists to engage in these discussions.

However, the division of responsibilities between nephrologists and primary care providers remains poorly defined. The nephrology community itself is divided on this issue, with 49% of nephrologists believing they should provide primary care to dialysis patients, and 40% feeling these responsibilities should fall outside their scope.^43^ This ambiguity was also reflected in our study, with some participants stating that reproductive counseling falls outside their scope of practice, instead emphasizing the role of gynecologists or primary care providers.

Nephrologists emphasized a lack of clinical guidelines, training, and resources that possibly contribute to inadequate reproductive health counseling. This identified subtheme echoes previous literature, such as a survey where 39% of nephrologists in the United States and Canada cited a lack of evidence in the field as a limiting factor for counseling. Nearly 15% of nephrologists did not provide any contraception or family planning counseling, with the most common reasons being lack of training (53.3%) and lack of personal knowledge (39.6%).^19^ These barriers were reiterated throughout our interviews, with reliance on self-education emerging as a subtheme.

Additionally, insufficient training in women’s health has been identified within medical education^24,44^ and was highlighted by a previous US national survey that reported 45.9% of graduated nephrology fellows answered that they had “some training, but not enough to feel confident” in kidney complications of pregnancy.^45^ This education gap is likely a contributing factor to the nephrologists’ expressed need for self-study, which can lead to inconsistent approaches to women’s health. Addressing this gap, whether through training exposure in fellowship or increased educational opportunities, remains important. Previous studies have shown that 67% of nephrologists identified continuing education materials as helpful in improving their ability to provide reproductive care,^19^ demonstrating an openness to education intervention.

Women with CKD are often managed by multiple specialists, increasing the chances of fragmented care.^46^ Our study participants highlighted challenges in interdisciplinary collaboration, particularly when providers were outside their institution. In response to this barrier, nephrologists stressed a patient’s role as the center of communication between specialists. It is important to contrast physicians’ expectations with patients’ experiences in terms of this issue. In a previous study, interviewed patients also reported feeling like the intermediary between specialties, associated with feelings of confusion and frustration.^18^ Any potential disconnect between the physician and patient in terms of interprofessional communication could be addressed by establishing clear expectations early on.

Multidisciplinary care models, including integrated obstetrics-nephrology clinics, are a promising approach to addressing these challenges. These approaches have been shown to improve patient outcomes and experiences.^47,48^ In particular, the expertise of fertility specialists, combined with nephrologists’ understanding of kidney-specific risks, can contribute to developing integrated care models that address the multifaceted needs of women with CKD. However, access remains a barrier: in 2019 only 12% of surveyed nephrologists in the United States and Canada reported access to such clinics.^19^

Expanded guidelines offer another possible approach to increased reproductive health in women with kidney disease. In one study, 83% of nephrologists indicated that interdisciplinary guidelines would enhance their ability to counsel and manage reproductive health and contraception in CKD patients, aligning with our identified themes of lack of guidelines and fragmentation of care.^19^ In parallel, technologies such as electronic health record prompts and AI-driven risk stratification algorithms could be leveraged to identify patients at elevated reproductive risk and prompt timely counseling discussions.^49,50^ In settings with time or comfort barriers, these tools can enhance patient care while preserving clinical judgment.

Chronic disease affects the patient in all dimensions, aside from just physical.^51,52^ A systematic review found that the pregnancy decisions of women with CKD can be emotionally complicated by health risks, family burdens, and social desires.^25^ These multifactorial impacts were echoed in our analysis, where nephrologists emphasized the importance of a holistic approach that prioritizes empathy, shared decision making, and comprehensive care planning. Previous research has demonstrated that incorporating these elements into care improves patient outcomes and satisfaction.^53,54^ Empowering patients through comprehensive counseling and a multidisciplinary approach can help address the multifactorial challenges associated with CKD.

Based on the themes and subthemes identified in the interviews, strategies and action items were established across 3 key domains to improve contraceptive use and reproductive health in women with kidney disease: (1) nephrologist training, (2) clinical practice, and (3) physician–patient communication. Suggested actions for each domain are outlined in Box 2. Addressing these domains may help improve reproductive health outcomes and overcome barriers for women with kidney disease.

**Figure 2.**
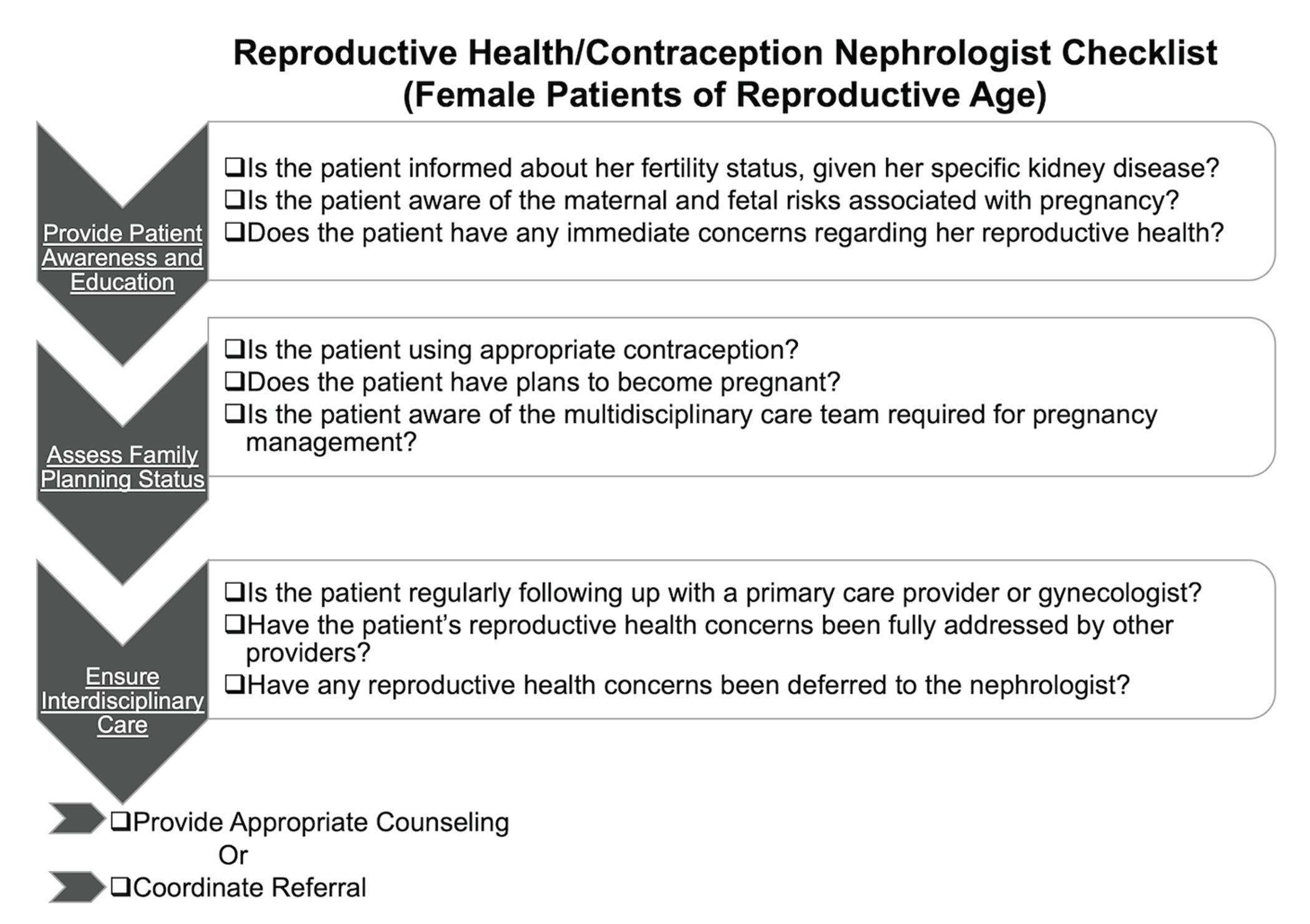
Suggested checklist for brief contraception and reproductive health assessment in females of reproductive age.

Drawing directly from the current study’s findings with participating nephrologists, a checklist was developed as a practical tool to support more consistent and comprehensive reproductive health counseling in nephrology practice. However, it has not yet been piloted or evaluated for its psychometric properties. The current version reflects provider perspectives, but incorporating patient input in future iterations may improve its effectiveness. The checklist, seen in Figure 2, is structured into 3 domains: (1) patient awareness and education, (2) family planning status, and (3) interdisciplinary care. Each domain includes 3 guiding questions for nephrologists to address. If a domain remains unaddressed based on the patient’s situation and responses, the checklist provides resolution options of (1) offering appropriate counseling or (2) coordinating a referral.

This checklist is designed to serve as a general reminder to briefly address key topics related to reproduction and contraception, particularly for nephrologists who may not frequently encounter female patients of reproductive age. By providing this tool, the aim is to bridge potential gaps in communication between providers and patients while encouraging a more consistent method of addressing these issues in a nephrology context. Potential future steps for refinement and validation include its implementation in nephrologists’ offices as a cognitive aid, followed by an evaluation of its utilization and impact on counseling rates.

Although practice-level interventions are essential, placing the responsibility solely on individual providers risks overlooking structural barriers. Systemic factors—such as limited time during clinical encounters, productivity demands, and lack of reimbursement mechanisms—constrain reproductive counseling in nephrology. Improving contraceptive use among women with CKD will likely depend on a multipronged approach that combines clinician education, institutional support, and policy that incentivize comprehensive reproductive care as a routine aspect of nephrology practice. Standardized documentation and quality metrics may help promote more consistent practice. However, such measures must be implemented thoughtfully to avoid unintended consequences, particularly in underresourced settings or when patients decline contraception for personal reasons.

There are a few limitations to our study. Given the recruitment method, there is a potential for selection bias because the participants may disproportionately represent nephrologists with a pre-existing interest in reproductive health. Additionally, most participants were early-career academic nephrologists, limiting generalizability to private or community-based settings where the identified barriers are likely to be even more pronounced. Further research, including a more diverse range of nephrologists alongside additional providers such as fertility experts, is warranted to expand on these findings. Incorporating perspectives from patients would also help align strategies with lived experience.

In conclusion, this study offers novel insights into nephrologists’ perspectives and experiences regarding contraceptive counseling and reproductive health management for women with CKD. Several key barriers emerged. Limited exposure to patients of reproductive age and lack of integration into nephrology practice contribute to provider discomfort. This discomfort is further compounded by the absence of clear guidelines and insufficient formal training, requiring providers to self-educate. Fragmentation of care also presents a significant obstacle, often necessitating reliance on patients to bridge communication. Despite these challenges, providers recognize the importance of holistic care, emphasizing empathy and shared decision making. These findings underscore the previously reported gaps in reproductive health management for women with CKD, highlighting the need for targeted interventions. Addressing the identified domains can allow patients to make more informed decisions, increase appropriate contraceptive use, reduce unplanned pregnancies, and ultimately enable the nephrology community to better support women with kidney disease.

## Supplementary Material

1 Supplementary File (PDF)
**Item S1:** Interview guide and focus group questions.

## Figures and Tables

**Table 1.** Participant Demographics and Clinical Characteristics (n = 25)

	Value
Age, y	40 [35-66]
Sex	
Male	10 (40%)
Female	15 (60%)
Years of practice since fellowship	7 [4-35]
Clinical scope	
General nephrology	17 (68%)
Transplant nephrology	8 (32%)
Current practice setting	
Academic	19 (76%)
Private	1 (4%)
Hybrid	5 (20%)
Patient population	
Adults	23 (92%)
Pediatrics	1 (4%)
Both	1 (4%)

Values are given as number (percentage) for categorical variables and as median [IQR] for continuous variables.

## PLAIN-LANGUAGE SUMMARY

Women with kidney disease can face challenges with fertility, sexual health, and menstruation. Pregnancy is often riskier for these women due to complications such as high blood pressure and preterm birth. Despite these risks, the use of birth control among women with kidney disease remains low. This study looked at the experiences of nephrologists in providing contraception and reproductive health counseling for their female patients. The interviews revealed that many nephrologists feel uncomfortable discussing these topics due to a lack of training and the absence of standardized guidelines. Communication between different health care providers involved in a patient’s care, such as gynecologists and primary care doctors, is also often limited. These challenges can make it harder for women to make informed decisions about contraception and family planning. There is a need for better training and resources for nephrologists so they can improve reproductive health counseling and help women make informed choices about contraception.

## Box 1. Illustrative Quotations by Themes Generated From Nephrologists’ Interviews

Physician Discomfort Regarding Discussion of Contraception and Reproductive Health

- Reliance on Patient-Initiation
- Hesitation With Counseling
- Uncertainty About Scope of Practice
- “Unless they [patients] bring up the topic, generally, it’s not on the list of things we [nephrologists] would like to discuss.” (39, general)
- “Only if patients bring it up, then I will address it, but I think over the last 6 months, I can’t think of one woman who brought it up. Men will bring it up, and I’ll talk about it if there’s medications that effect it, and then I usually refer them to their primary care, again, if they have ED [erectile dysfunction] or other things.” (47, transplant)
- “Quite honestly, in 12 years of experience, nobody has ever brought this up to me, and I usually don’t take care of this. It’s something that’s left to the primary care physician or to the OB [obstetrician].” (44, general)
- “So if I need to start some medication that could potentially be critical for [reproduction], then I would talk to them. Otherwise, it’s not even a conversation.” (40, general)
- “A general practitioner may not recognize that as much as a nephrologist does, but the nephrologist may also kind of forget to bring it up because we’re so used to having patients who aren’t able to conceive.” (37, general)
- “I think it’s the lack of knowledge, not just from the patient side but from the physician side. We are not really equipped to talk about these things.” (40, general)
- “If they need contraception, I am not confident enough on the type of contraception to recommend because there’s so many options there now. So I end up saying, ‘Oh yeah you going to need some contraception. You will need to talk to [another provider] to discuss further.’ And then I’ll end there.” (40, general)
- “I think that many [physicians] out of a lack of knowledge, fear, comfort, will take what they perceive to be a more conservative course and refuse to prescribe a daily combined oral contraceptive to [a patient with CKD] with well-controlled hypertension, even though the evidence probably suggests that it’s okay.” (37, general)
- “I am actually better at [reproductive health discussions] with men than I am with women, which is embarrassing for me as a cis woman … So I’m terrible at it … I think there is discomfort on both sides [patient and provider].” (37, general)
- “I’m embarrassed to say that I don’t really talk about it a lot. In fact, I wouldn’t be surprised if I’ve only done it, like, zero or one times in my practice.” (38, transplant)
- “You can ask other nephrologists, but I doubt if anybody is interested to be involved in contraceptives, or reproductive health—that, we leave to somebody else who has more expertise and is able to handle those matters.” (66, general)
- “I’ll be honest I don’t think nephrologists should be the ones doing this counseling. I think nephrologists should be the ones making sure that it has happened, but my job is to take care of your kidney disease not to take care of your contraception.” (37, general)

Insufficient Training and Inadequate Guidelines Regarding Contraception and Reproductive Health

- Paucity of Guidelines
- Limited Exposure
- Reliance on Self-Education
- “Not much literature and clear-cut guidelines in terms of what is more effective in which situation. I mean we [physicians] all know that as chronic kidney disease progresses, the chances of infertility increase or— but there is not much data on how much is the percentage with CKD, what is the difference with different etiologies. So there’s a lot of lack of data and what is effective and what is not effective.” (39, general)
- “I’m not sure if we have any guidelines. [Education on the subject matter] is very minimal.” (66, general)
- “[Nephrologists] all very poorly educated on the pros and cons of [contraceptive use] at different stages of CKD.” (44, general)
- “As a nephrologist, I don’t get updated about the newest available contraceptive pills or the side effects.” (66, general)
- “I know in our med student courses, we [physicians] talk about [sexual and reproductive health] very briefly because we’re given 2 weeks to talk about everything clinical in kidneys.” (35, transplant)
- “From a training perspective, I may have had some lectures in residency or even medical school on [reproductive and sexual health among CKD patients] … but it’s pretty much all been independent study … to get to the point where I feel semi-comfortable … but definitely not an expert … I think if there were resources … or reference manuals, then [physicians] would be better able and better equipped to [discuss with patients] … I think guidelines with flow charts or different diagrams … with a case style teaching would be helpful.” (36, general)
- “We need resources or education sessions because [reproductive and sexual health] isn’t something that’s been taught very well to nephrologists, mainly because we just don’t see it that often and we don’t usually treat women under 30 who want to get pregnant. [Women under 30 who want to get pregnant] is not a very common demographic of kidney disease patients, so it hasn’t been very prevalent … I feel like education, materials, or lectures is definitely something that’s needed.” (41, general)
- “[Sexual and reproductive health] have to be incorporated into the curriculum during training. If [nephrologists] are not sensitized during their training, they are not going to get sensitized ever. So the basics of reproductive health and its impact on Illustrative Quotations by Themes Generated From Nephrologists’ Interviews kidney disease, what are the dos, what are the don’ts, and what are things [nephrologists] need to talk about should be heavily discussed during the training of a nephrology fellow.” (41, general)
- “I feel like a lot of what [physicians] know [about sexual and reproductive health in CKD patients] is probably outdated and probably not as fact-based as I wished it was.” (39, general)
- “I did not have any specific training. I built my own training and practice … like, I’m going to make sure this is a part of my practice, so when I see women in their childbearing age group, I know what to do.” (40, transplant)
- “Either [nephrologists] train themselves because they are expertise deficient in the areas of reproductive and sexual health, or they refer the patient elsewhere.” (66, general)

Lack of Interdisciplinary Coordination Regarding Contraceptive Use and Reproductive Health

- The Patient as an Intermediary
- Fragmentation of Care
- “If you’re all in the same health system, it’s easier to just talk to each other and collaborate. But sending the notes, I don’t know if they get read or not, but that’s what we do. And then through the patient, basically, I think the patient becomes the source of communication.” (47, transplant)
- “[Physicians] have to be proactive to make sure that doctor’s notes make it to that patient’s other physicians or health portals or however they like to receive their specialist notes, and … tell the patient they have to be proactive as well. [Physicians] mostly communicate via just shared notes … making the patient the center and [the bridge] between the specialties.” (41, general)
- “It’s helpful if [physicians] share a medical record because that allows to very easily share in writing discussions and notes. I think communication is really the biggest [barrier]. It is a clear delineation of who’s responsible for what and [facilitates] discussions when things are not going as expected.” (37, general)
- “Everybody works in their own specialties. Sometimes it’s hard to even get the progress note because it’s not an interconnected system … Most of the time, if things are stable, you just presume the other providers are taking care of matters themselves.” (50, general)
- “I am not doing a great job with collaboration. So either I will say, ‘Go talk to your OB about it,’ or I will send [the patient] to my OB nephrology colleague, who may send [the patient] to another person.” (40, general)
- “So when we form that team, you know, it may be an institution or maybe outside the institution, having that to-go person, you know, and that person to collaborate with and know that you can communicate with is so crucial. It makes the work easier. It makes communication better, and it definitely amplifies patient care.” (40, transplant)
- “If the providers are within a different health care organization, then I think it just needs to be a little bit of an extra effort on everyone’s part to make sure that everyone’s communicating well.” (45, general)
- “[Barriers] are fragmentation of care, lack of knowledge, lack of ability … Care coordination can be complex.” (37, general)
- “If [physicians] can communicate with each other, like if they have any questions, and reach out to each other, I think that would be better because that way we can improve the patient care and work together. Unfortunately, in the real world, [communication] doesn’t happen.” (50, general)
- “Out of network, it’s sometimes hard to get to the provider, and you have to jump through a lot of hoops … and it may not happen in a timely fashion … so you may forget and move on.” (50, general)

Need for Holistic and Patient-centric Care

- Comprehensive and Sustained Approach
- Shared DecisionMaking
- “A patient’s mental health can impact kidneys, and [kidneys] can impact mental health.” (37, general)
- “You [can’t always] focus on the clinical side of the outcome. You have to see the emotional part of their body and their health, too.” (50, general)
- “You [the physician] want to always encourage and give all the information and ensure that that person leaves your office, you know, even if they didn’t hear the answer that they wanted, they have some sort of relief and they are not anxious, you know, about what is to come.” (40, transplant)
- “The conversation I think needs to be more emphatic and more recurrent to make sure that the patient understands the implications [of chronic kidney disease on reproductive and sexual health].” (35, general)
- It’s important to build a relationship of communication and trust with your patient, where you can work closely with them. The more trust your patient has with you, the more they communicate, the more confident you will be with them, and the more comfortable they will be with you and trust in your judgment. (50, general)
- “Our [physician] role involves establishing a relationship, securing trust in a relationship, and, you know, ensuring compassion in a relationship so that when you talk to the patient and say, ‘Hey, I need you to check this thing out—or check this link out. Go and tell me what you think.’” (40, transplant)
- “You [physician] just take 2 minutes … to start that conversation. You trust in your patient and empower them to ask questions and look up various things on various websites.” (40, transplant)

## Box 2. Suggested Strategies and Actions in the Field of Nephrology

Nephrologist Training

- Consider the nephrologist’s role in contraception and reproductive health counseling.
- Stay up to date on relevant research surrounding reproductive health and kidney disease.
- Encourage involvement in research regarding the interplay between CKD and reproductive health.
- Interdisciplinary sessions are recommended primarily for high-risk or complex cases—such as pregnancy in the setting of advanced CKD or kidney failure—where collaborative management is essential to optimizing patient outcomes.
- Provide continuing medical education (CME) credit courses that help nephrologists better understand fertility and CKD.^a^
- Increase exposure to educational sessions focusing on correlations between fertility and CKD.^a^
- Provide training sessions exploring family planning options for patients with kidney disease.^b^

Clinical Practice

- Share notes and plans of care between nephrologists, gynecologists, and primary care physicians.
- Increase utilization of MyChart by specialists and patients.
- Encourage chart review for out-of-network specialists.
- Verify whether the patient is following up with other specialists.
- Verify whether the patient has received reproductive health and contraceptive counseling from other providers.
- Establish a referral plan for complex patients to specialists in reproductive health and kidney disease.
- Connect patients with resources that provide detailed information on fertility with kidney disease.^c,d^
- Consistently verify any changes in patients’ reproductive health or contraception.
- Develop a routine habit of briefly addressing the following in all patients of reproductive age:
  - Contraceptive use status
  - Following-up obstetrician/primary care
  - Awareness of pregnancy possibilities given kidney status
  - Any plans for pregnancy
  - Patient concerns regarding sexual dysfunction, menstrual abnormalities, and breastfeeding
  - Referral to an appropriate specialist for reproductive health discussions (if applicable)

Physician–Patient Communication

- Determine baseline knowledge regarding reproductive health with each patient instead of assuming what a patient knows.
- Provide detailed explanations to patients about fertility and adverse pregnancy outcomes associated with kidney disease.
- Set realistic expectations for the patient’s role in bridging communication between their health care team.
- Encourage patients to ask questions.
- Encourage patients to actively participate in decisions regarding their care.
- Acknowledge the widespread impact of CKD beyond the kidney.
- Be honest with patients.
- Build trust with patients.
- Promote shared decision making.
- Encourage patients to better understand their disease using reputable third-party online resources. Examples of resources include:
  - National Kidney Foundation (NKF): Patient Education Library.
  - Kidney Care UK: Patient information booklets (Women’s Health)

Abbreviation: CKD, chronic kidney disease.

^a^Nephrology professional organizations such as the American Society of Nephrology (ASN), the National Kidney Foundation (NKF), and the European Renal Association (ERA) are well-positioned to lead these efforts by offering board-reviewed continuing medical education (CME) courses that focus on the intersection of reproductive health and CKD.

^b^Appointing nephrology fellowship directors who have pursued advanced training in reproductive health could help embed this content into fellowship curricula and increase early-career exposure. Such efforts would not only improve clinical competency but also align with current recommendations for integrated, patient-centered care.

^c^If local expertise is limited or unavailable, nephrologists are encouraged to refer patients to national and international resources that provide reputable, evidence-based information. Recommended sources include the National Kidney Foundation (NKF), the American Society of Nephrology (ASN), the International Society of Nephrology (ISN), and the United Kingdom (UK) Kidney Association, all of which offer patient-oriented educational materials.

^d^Additionally, peer-reviewed platforms such as UpToDate and the patient information sections of academic medical centers serve as accessible and reliable resources to support patient education in the absence of local subspecialty care.
