# Supplementary material for: Contraceptive Use and Reproductive Health in Women With CKD: A Qualitative Study of Nephrologists in the United States
Source: Am J Kidney Dis. Author manuscript; Available in PMC 2026 Jun 15. (PMC13267251; doi:10.1053/j.ajkd.2025.07.007)
Supplement: 1 — Supplementary File (PDF) Item S1: Interview guide and focus group questions. [file NIHMS2183437-supplement-1.pdf]

Item S1. Interview guide and focus group questions.

1. What are your thoughts on counseling women with CKD on reproductive health?
   a. How can it be effectively provided?
   b. What topics should be discussed?
   c. How often should women receive it?

2. How do you conduct reproductive counseling for women with CKD?
   a. What challenges have you faced?
   b. How comfortable are you discussing sexual dysfunction?
   c. How do you evaluate your effectiveness?

3. What reproductive concerns are common for women with CKD?
   a. Menstrual irregularities?
   b. Fertility issues?

4. What factors should providers consider when women with CKD consider pregnancy?
   a. Timing?
   b. Kidney function status?
   c. Potential risks (i.e., fetal, and maternal)?
   d. Any others?

5. What are your thoughts about women with CKD who intend to breastfeed?
    a. Safety?
    b. If yes, please explain.
    c. If no, why not?

6. What concerns do women with CKD express about contraceptive use?
    a. What misconceptions are common?

7. What obstacles do women with CKD face in accessing contraceptives?

8. What barriers do providers encounter that limit effective contraceptive counseling?
    a. How can these barriers be overcome?

9. How do you counsel women with CKD on contraceptives?
    a. What helpful tools have you found?
    b. What guidelines do you follow?
    c. What is your role in the patient's decision-making?

10. What risks are associated with combined oral contraceptive use in women with CKD?
    a. How aware are patients of these risks?

11. What are the benefits of combined oral contraceptive use for women with CKD?
    a. How are patients made aware of the benefits?

12. What contraceptive methods do your patients tend to prefer?
    a. How do you balance these preferences with the patient's medical condition?

13. How does the medical history of a woman with CKD affect the likelihood of her receiving counseling on reproductive health/contraceptive use?

14. How is counseling on reproductive health/contraceptive use influenced by different treatment stages of CKD (non-dialysis, dialysis, post-transplant)?

15. What best practices promote effective multidisciplinary teamwork among providers for managing reproductive health/contraceptive use among women with CKD?
    a. How do you ensure collaboration among different providers?

16. What factors influence your confidence in managing reproductive health/contraceptive use among women with CKD?
    a. What specific training have you received related to reproductive health and kidney disease?

17. What strategies can help nephrologists better provide reproductive health/contraceptive use counseling?
    a. What resources might be helpful?
